# Prognostic Relevance of the Eighth Edition of TNM Classification for Resected Perihilar Cholangiocarcinoma

**DOI:** 10.3390/jcm9103152

**Published:** 2020-09-29

**Authors:** Hans-Michael Hau, Felix Meyer, Nora Jahn, Sebastian Rademacher, Robert Sucher, Daniel Seehofer

**Affiliations:** 1 Department of Visceral, Transplantation, Vascular and Thoracic Surgery, University Hospital of Leipzig, 04103 Leipzig, Germany; fmeyer94@icloud.com (F.M.); sebastian.rademacher@medizin.uni-leipzig.de (S.R.); robert.sucher@medizin.uni-leipzig.de (R.S.); daniel.seehofer@medizin.uni-leipzig.de (D.S.); 2 Department of Visceral, Thoracic and Vascular Surgery, University Hospital and Faculty of Medicine Carl Gustav Carus, Technische Universität Dresden, 01307 Dresden, Germany; 3 Department of Anesthesiology, University Hospital of Leipzig, 04103 Leipzig, Germany; nora.jahn@medizin.uni-leipzig.de

**Keywords:** perihilar cholangiocarcinoma, Klatskin tumor, liver resection, TNM classification, 8th edition, AJCC, prognostic model

## Abstract

Objectives: In our study, we evaluated and compared the prognostic value and performance of the 6th, 7th, and 8th editions of the American Joint Committee on Cancer (AJCC) staging system in patients undergoing surgery for perihilar cholangiocarcinoma (PHC). Methods: Patients undergoing liver surgery with curative intention for PHC between 2002 and 2019 were identified from a prospective database. Histopathological parameters and stage of the PHC were assessed according to the 6th, 7th, and 8th editions of the tumor node metastasis (TNM) classification. The prognostic accuracy between staging systems was compared using the area under the receiver operating characteristic curve (AUC) model. Results: Data for a total of 95 patients undergoing liver resection for PHC were analyzed. The median overall survival time was 21 months (95% CI 8.1–33.9), and the three- and five-year survival rates were 46.1% and 36.2%, respectively. Staging according to the 8th edition vs. the 7th edition resulted in the reclassification of 25 patients (26.3%). The log-rank *p*-values for the 7th and 8th editions were highly statistically significant (*p* ≤ 0.01) compared to the 6th edition (*p* = 0.035). The AJCC 8th edition staging system showed a trend to better discrimination, with an AUC of 0.69 (95% CI: 0.52–0.84) compared to 0.61 (95% CI: 0.51–0.73) for the 7th edition. Multivariate survival analysis revealed male gender, age >65 years, positive resection margins, presence of distant metastases, poorly tumor differentiation, and lymph node involvement, such as no caudate lobe resection, as independent predictors of poor survival (*p* < 0.05). Conclusions: In the current study, the newly released 8th edition of AJCC staging system showed no significant benefit compared to the previous 7th edition in predicting the prognosis of patients undergoing liver resection for perihilar cholangiocarcinoma. Further research may help to improve the prognostic value of the AJCC staging system for PHC—for instance, by identifying new prognostic markers or staging criteria, which may improve that individual patient’s outcome.

## 1. Introduction

Perihilar cholangiocarcinoma (PHC) is a relatively uncommon disease, but the treatment of this cancer is still challenging. Various treatment options and combinations consisting of chemotherapy and/or radiotherapy did not result in marked improvements in long-term patient outcomes, and operative resection remains the only possible curative therapy for these patients [1,2]. Due to complex anatomy and tumor configuration, however, surgery for PHC can be technically ambitious and may therefore represent a surgical challenge for hepato-pancreato-biliary surgeons. Hepatectomy with lymph node dissection and en bloc resection of the extrahepatic duct and caudate lobe, together with resection of the portal vein and/or hepatic artery when indicated, is an aggressive approach for PHC that can result in disease-free and overall survival [3,4,5]. Recent studies have reported five-year survival rates following curative-intent liver resection varying from 25% to 40% [1,3,6,7,8].

Due to clinically inapparent tumor progress, many patients show metastatic or locally advanced disease at first clinical presentation and, therefore, do not profit from resection [9,10,11,12,13].

However, there are various factors that may influence the prognosis of patients with PHC. This is why predicting long-term survival can be challenging, and accurate stratification of patient prognosis remains difficult [11,14,15]. In this context, physicians try to determine long-term survival by different prognostic tools that integrate the most relevant clinicopathological factors [6,7,14,16,17,18]. This includes the Bismuth-Corlette classification and the Blumgart T-stage system, which focus on determining resectability using preoperative imaging [7,19,20]. However, the American Joint Committee on Cancer (AJCC) staging system is the most common scheme for the evaluation of prognosis and consecutive treatment and for comparing outcomes between other centers [21].

The AJCC staging system has changed over the recent years: in the 6th edition (2003), all patients with extrahepatic bile duct tumors were included in one classification [22], whereas the 7th edition (2010) was the first system to differentiate between perihilar (proximal) and distal cholangiocarcinoma [23,24]. The recently published 8th edition of the AJCC staging manual now has implemented alterations to the T-, *n*-, and overall stage category classification schemes [16,20,21,23,25] (Table 1). In particular, the T4 disease category excludes bilateral second-order bile duct extension and is now defined as a tumor invading the main portal vein or its branches bilaterally, the common hepatic artery, or the unilateral, second-order biliary radicals with contralateral portal vein or hepatic artery involvement [26]. This new definition now implicates that T4 tumors are classificated as stage IIIB instead of IVA with a newly introduced stage IIIC (any T, N1, and M0). Furthermore, in the 8th edition, the lymph node staging (*n*-category) of patients with PHC was altered, with the N1- and N2-stage categories based on the counts of positive lymph nodes (N1: one to three involved positive regional lymph nodes and N2: four or more involved positive regional lymph nodes).

The current literature contains only a few studies that have utilized the recent AJCC 8th edition staging system for patients and, especially, those undergoing curative liver resection for PHC at high-volume hepato-pancreato-biliary (HPB) centers [25,27,28]. In addition, there are only a few data analyzing the prognostic accuracy of the 8th TNM edition of patient prognosis in patients undergoing curative-intent liver surgery for PHC in Western centers [25].

Against this background, the aim of the current study was to compare the prognostic accuracy and validation of the newly published tumor node metastasis (TNM) classification of the AJCC 8th edition with the 7th and 6th editions for patients with PHC following curative liver resection at a big Western HPB center. Specifically, we wanted to evaluate whether the new PHC classification provides improved distinction between tumor stages and a more accurate prediction of patient survival. We also wanted to evaluate survival rates with regards to biological tumor markers and pathological characteristics.

## 2. Methods

### 2.1. Study Population

This study included patients from a prospectively collected database at the University Hospital of Leipzig. The study was approved by the local ethical commission board from the University of Leipzig (AZ- EK: 243-14-14072014). The study included only patients with histological confirmation of PHC and curative intent liver resection.

Patients were excluded in our study because of the following reasons: patients undergoing explorative laparotomy and/or palliative bypass operation due to distant metastases and/or advanced disease, patients who underwent an R2 (macroscopic positive resection margins)- resection and/or local ablation, patients with a final pathology indicative of a diagnosis other than PHC, and patients with advanced unresectable diseases and/or poor liver function during the preoperative diagnostic workup.

### 2.2. Patient Management

Preoperatively, every patient received a diagnostic workup and tumor staging for a multidisciplinary decision.

This preoperative workup and staging including an assessment of medical history, physical examination, and cross-sectional imaging with computed tomography (CT) of the abdomen and chest, as well as magnetic resonance imaging (MRI) with magnetic resonance cholangiopancreatography (MRCP) of the liver at or before the initial referral (ideally prior to biliary drainage). The preoperative imaging was used to assess the local and biliary extent of the tumor and detection of distant metastases, such as the relationship and involvement of vascular structures. In cases of obstructive jaundice and/or cholangitis, a biliary drainage procedure ideally in the presumed remnant liver was performed. Endoscopic biliary drainage (EBD) was the preferred method, but percutaneous transhepatic biliary drainage (PTBD) was performed when EBD was not successful. A brush was typically used at the time of preoperative biliary drainage, but pathologic confirmation of malignancy was not required to proceed with surgical resection. Since the accuracy of preoperative cross-sectional imaging techniques has improved during the last years, the yield and rate of diagnostic laparoscopy has frequently decreased.

Patients considered to have an insufficient size and function of the future liver remnant underwent preoperatively a portal vein embolization (PVE). Neoadjuvant chemotherapy was administered in fewer than 10% of the study patients.

With some exceptions, unresectability of the disease was defined as the presence of extensive bilobular metastases or extrahepatic metastases, peritoneal dissemination, and poor patient’s condition and liver function, such as involvement and/or encasement of major vessel structures without the possibility for reconstruction.

The operative procedure consisted of extrahepatic bile duct resection in combination with complete en-bloc dissection of regional lymph nodes, including tissue of the hepatoduodenal ligament, the posterior surface of the head of the pancreas, and the common hepatic artery, followed by dissection of the hilar structures. Regional lymphadenectomy was carried out in all patients, and in cases of macroscopically abnormal interaortocaval and celiac lymph nodes, these areas were cleared. Extent and type of hepatectomy was selected due to the relative location and extent of the tumor according to the Bismuth-Corlette classification [19,29]. In cases of suspected macroscopic vascular invasion during the operation, resection and reconstruction of the portal vein and hepatic artery were carried out. Prior to December 2007, caudate lobe resection (CLR) was performed at the discretion of the surgeon. If, during the operation, the surgeon felt the tumor had infiltrated the caudate bile duct, CLR was performed to obtain a radical resection. However, from January 2008 onward, CLR was routinely performed with prophylactic portal vein resection and reconstruction combined with extended hepatectomy as part of the no-touch technique [3].

### 2.3. Data Collection

From patient’s medical records; data on standard demographics; and perioperative and clinicopathological characteristics, including gender, age, and American Society of Anesthesiologists (ASA) classification, such as preoperative comorbidities, were collected and analyzed. Furthermore, biochemical tests, including the analysis of serum levels of carcinoembryonic antigen (CEA), Cancer Antigen (CA) 19-9, and preoperative bilirubin levels, were performed.

With regards to treatment characteristics, the extent and type of liver resection, use of preoperative biliary drainage/endoscopic stenting, and preoperative portal venous embolization (PVE), such as the administration of neoadjuvant/adjuvant treatments, were assessed.

Based on the final pathologic reports, tumor-specific characteristics and the resection margin status (R0—microscopically negative and R1—microscopically positive), such as the number and site of harvested and metastatic lymph nodes, were evaluated.

### 2.4. Staging

Differences and definitions of the of the 6th, 7th, and 8th AJJC TNM staging classification systems were depicted in Table 1. Tumor stage and patient classification were determined for each patient separately according to the 6th, 7th, and 8th editions of the AJCC TNM staging system evaluating radiological scans, as well as pathological specimens. Therefore, four different and independent investigators, two experienced radiologists and two experienced pathologists, analyzed and classified each scan or specimen independently according to the different AJCC TNM staging systems. In the case of missing concordance between the investigators, a validation was performed by a third and independent investigator.

### 2.5. Statistical Analysis

Continuous variables were reported as mean/median values with interquartile range (IQR)/standard deviation depending on the normality of the distribution, whereas categorical variables were expressed as total counts and percentages (%).

For the analysis of the baseline data, the appropriate statistical tests, including the chi-square test, Student’s *t*-test, analysis of variance (ANOVA), Kruskal-Wallis, and/or Wilcoxon-Mann-Whitney test, were applied.

According to previous definitions, overall patient survival (OS) was defined from date of liver surgery until patient death or last follow-up for living patients.

Survival rates were calculated using the Kaplan–Meier method, and a log-rank test was applied to compare survival curves.

Stepwise multivariate survival analysis was performed using a Cox proportional hazards regression model to check associations between the tumor stage and OS. In the survival analysis, clinicopathological and demographic variables resulting in a statistically significant value (*p* < 0.05) in the univariate analysis were entered into the multivariate analysis to assess their independency.

The coefficients from the Cox models were reported as hazard ratios (HRs) with corresponding 95% confidence intervals (CIs).

The area under the curve (AUC) of the receiver operating characteristic curve (ROC) was used to calculate the discriminative power and prognostic accuracy of each edition of the TNM staging systems. For assessment of the calibration of our staging models, we used the Hosmer–Lemeshow chi-square test to determine the goodness of fit. Patients with ongoing follow-up were censored at the last time point of examination.

All data were analyzed by using SPSS software (SPSS Inc., Chicago, IL, USA, version 25), and a *p*-value < 0.05 was considered statistically significant.

## 3. Results

### 3.1. Baseline Characteristics

In total, 189 consecutive patients underwent surgery between 2002 and 2019. In 95 patients, liver resection was performed with curative intention, whereas 94 (49%) patients underwent an explorative laparotomy due to peritoneal carcinosis, reduced liver function, or major tumor extension.

The baseline characteristics of our patients are illustrated in Table 2. The median age of the patients at the time of surgery was 64.9 ± 10.2 years, and 54.7% (*n* = 52) of the patients were male. The mean preoperative total bilirubin levels before operation were 44.2 ± 11.1 µmol/l. Preoperatively, biliary drainage was performed in 73 patients (77%), with endoscopic biliary drainage as the most common procedure (*n* = 64; 67%). PVE was preoperatively carried out in 21 patients (*n* = 22.1%).

The most common types of Bismuth-Corlette classification were Type IV (*n* = 49; 51.6%), but 6 patients (6.3%) had type I, 8 patients (8.4%) had type II, and 23 patients (24.2%) had type III. In total, 9 patients (9.5%) received neoadjuvant chemotherapy and 26 patients (27%) received adjuvant chemotherapy.

Major hepatectomy was carried out in 91 patients (96%), with left (*n* = 16; 17%) and right (*n* = 54; 57%) trisectionectomies most commonly performed. In this context, portal vein resection (PVR) was carried out in 64 patients (67.3%), whereas a caudate lobe resection was performed in 67 patients (70.5%).

Postoperatively, total complications were observed in 69 patients (72%), with major complications (>III according to Clavien-Dindo Classification) in 55% (*n* = 54 patients). The 90-day mortality was 9.4% (*n* = 9 patients)

### 3.2. Pathologic Findings

All resected specimens were confirmed as adenocarcinomas. R0 resection margins were achieved in 68 patients (71.6%) (Table 2). Microvascular invasion was found in 24 (25.3%) patients and perineural invasion in 59 (62.1%) patients. The invasion of small lymphatic vessels was observed in 68 patients (71.6%).

Pathology revealed poorly differentiated tumors in most patients (*n* = 48, 50.5%) and either well-differentiated (*n* = 5, 5.3%) or moderate tumors (*n* = 42, 44.2%) in the remainder. Almost one out of three patients had lymph node metastases (*n* = 27, 28.4%), and approximately one in ten patients had metastatic disease at diagnosis (*n* = 8, 8.8%).

The median number of lymph nodes (LN) harvested was four (IQR 3-8), and the median number of metastatic lymph nodes (MLN) was one (IQR 0-2).

### 3.3. Stage Migrations

In the 6th edition of the AJCC staging system, 5 (5.3%) patients were grouped in TNM stage Ia, 18 (18.9%) in stage Ib, 38 (40.4%) as stage IIa, 20 (21.1%) as stage IIb, 6 (6.3%) as stage III, and 8 (8.4%) patients as stage IV.

In contrast, the 7th edition of the AJCC staging categorized 5 (5.3%) patients as TNM stage I, 52 (54.7%) as stage II, 4 (4.2%) as stage IIIa, 18 (18.9%) as stage IIIb, 5 (5.3%) as stage IVa, and 11 (11.5%) patients as stage IVb.

The 8th edition of the AJCC staging categorized 5 (5.3%) patients as stage I, 52 (54.7%) patients as stage II, 4 (4.2%) as stage IIIa, 3 (3.1%) as stage IIIb, 18 (18.9%) as stage IIIc, 5 (5.3%) as stage IVA, and 8 (8.4%) patients as stage IVB.

In Table 3, the different stages and their changes of the 6th, 7th, and 8th editions of the AJCC staging system are presented.

When comparing the 6th edition with the 7th edition, a total of 45 (47.4%) patients changed stages based on the changes in the major group definitions (Table 3). However, after comparison of the 7th with the 8th edition, a total of 25 patients (26.3%) were reclassified when considering the substages and 23 patients (25%) when considering only the major stages.

Compared to the 7th edition, the 8th TNM edition staging downstaged 6 patients (6.3%) and upstaged 18 patients (18.9%).

According to the lymph nodes, a total of 18 patients (18.9%) with N1 disease (corresponding stage IIIb in the 7th edition) in the 7th edition moved to stage IIIc (*n* = 15 patients; metastasis in one-three lymph nodes) or IVa (*n* = 3 patients; >four or more positive lymph nodes) in the newly 8th edition.

With regards to the T classification, most patients in the 7th T4 category (*n* = 5 patients; corresponding stage IVa) moved to stage IIIb (*n* = 3 patients) or IIIc (*n* = 1 patient) in the 8th edition.

### 3.4. Survival Analysis

The median follow-up time of patients after hepatic resection was 4.3 ± 2.9 years.

The overall median survival of the entire patient cohort was 21 months (95% CI 8.1–33.9), with survival rates after hepatic resection at one, three, and five years of 68.4%, 46.1%, and 36.2%, respectively. Tumors recurred in 48 (51%) patients after a median disease-free survival time of 32 (95% CI: 22.8–45.4) months.

We tested several clinical and pathological factors on the OS, as shown in Table 4. We found that the OS was significantly longer in female patients (*p* = 0.02), patients with no extrahepatic distant metastases (*p* < 0.001), those with negative resection margins (*p* = 0.003), those aged < 65 years (*p* = 0.03), those with good/moderate tumor differentiation (*p* = 0.01), and patients with no microvascular invasion (*p* < 0.01).

More importantly, lymph node metastasis was strongly associated with worse outcomes. Patients with no lymph node involvement had a five-year overall survival of 33.3%, compared with 24.3% and 0% for patients with one–three and >three metastatic lymph nodes (*p* < 0.01).

Further analysis showed that, in patients undergoing PVR, no statistical better outcome (*p* > 0.05) was achieved, whereas, in patients undergoing caudate lobe resection, five-year OS rates of 28.7% were compared with 17.1% for those who could not (*p* = 0.004) be observed.

The multivariate analysis (Table 5) revealed that gender (male vs. female; HR = 2.6, 95% CI: 1.4–4.9; *p* < 0.01), age (>65 years vs. <65 years; HR = 1.9, 95% CI: 1.2–3.2; *p* = 0.04), margin status (R1 vs. R0; HR = 2.1, 95% CI: 1.2–3.5; *p* = 0.01), distant metastases (M1 vs. M0; HR = 4.2, 95% CI: 1.1–10.8; *p* = 0.002), metastatic lymph nodes (1–3 MLN vs. 0 MLN, HR = 1.1, 95% CI: 0.5–2.1; *p* = 0.04; > 3 MLN vs. 0 MLN; HR = 3.5, 95% CI: 1.4–9.1; *p* < 0.01), tumor differentiation (G3 vs. G1; HR = 7.9, 95% CI: 1.1.-58.1; *p* = 0.04), and caudate lobe resection (no vs. yes; HR 2.2, 95% CI: 1.2–4.1; *p* < 0.01) remained associated as independent predictors of the OS.

### 3.5. Survival across Stages

Overall, an unbalanced distribution was observed that favored stage IIa (40%) in the 6th edition and stage II (54.7%) in 7th and 8th editions. Stage-specific survival rates due to the different TNM editions of the AJCC staging system are shown in Table 6**.**

Due to the 6th, 7th, and 8th editions of TNM classification, the median overall survival rates were as follows: stage I (6th, 7th, and 8th editions: 58 months (95% CI: 48.8–67.2) vs. 62 months (95% CI: 34.4–89.6) vs. 62 months (95% CI: 34.4–89.6)); stage II (6th, 7th, and 8th editions: 37 months (95% CI: 21.4–52.6) vs. 43 months (95% CI: 19.5–66.5) vs. 43 months (95% CI: 19.5–66.5)); stage III (6th, 7th, and 8th editions: 28 month (95% CI: 8.8–47.2) vs. 37 (95% CI: 1.6–72.4) vs. 38 (95% CI: 24.6–51.4)); and stage IV (6th, 7th, and 8th editions: 12 months (95% CI: 0–24.5) vs. 22 (95% CI: 7.6–36.4) vs. 16 (95% CI: 10.1–21.9)), respectively (*p*-values between stages in the 6th edition = 0.039 vs. in the 7th = 0.010 vs. in the 8th = 0.002).

**Figure 1:**
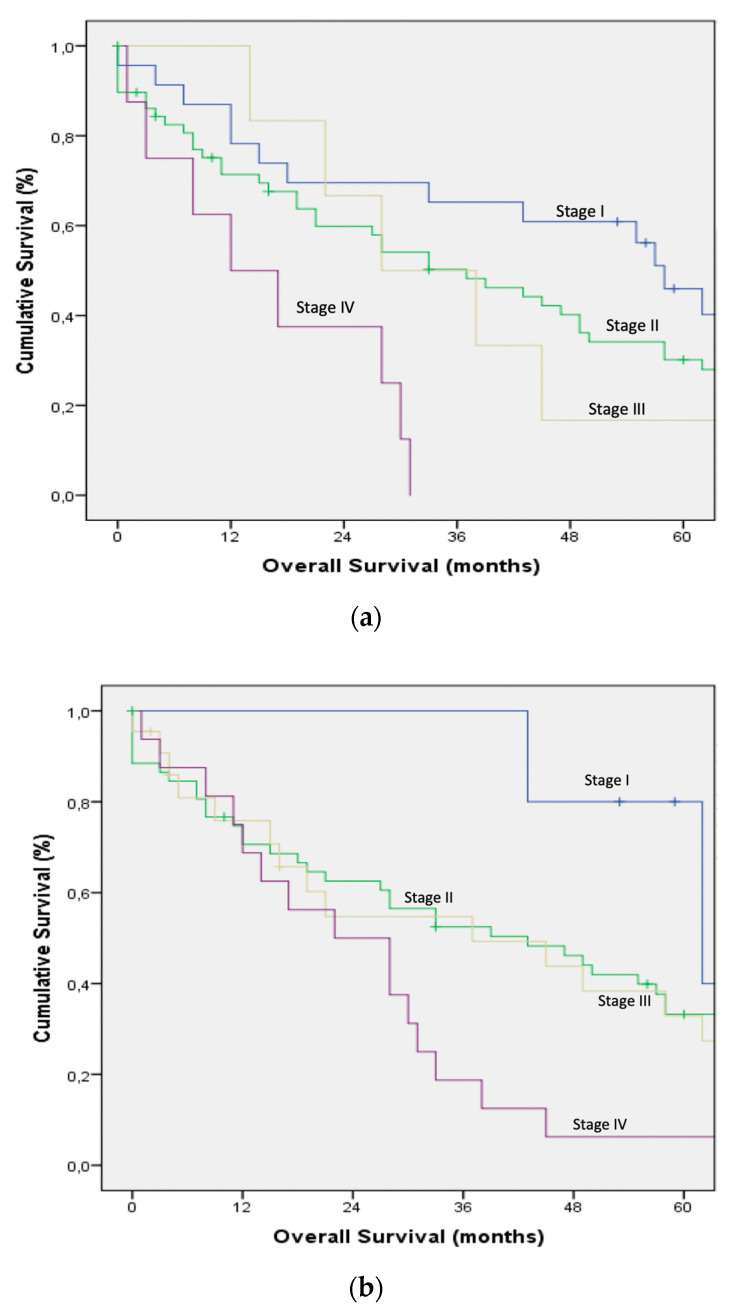

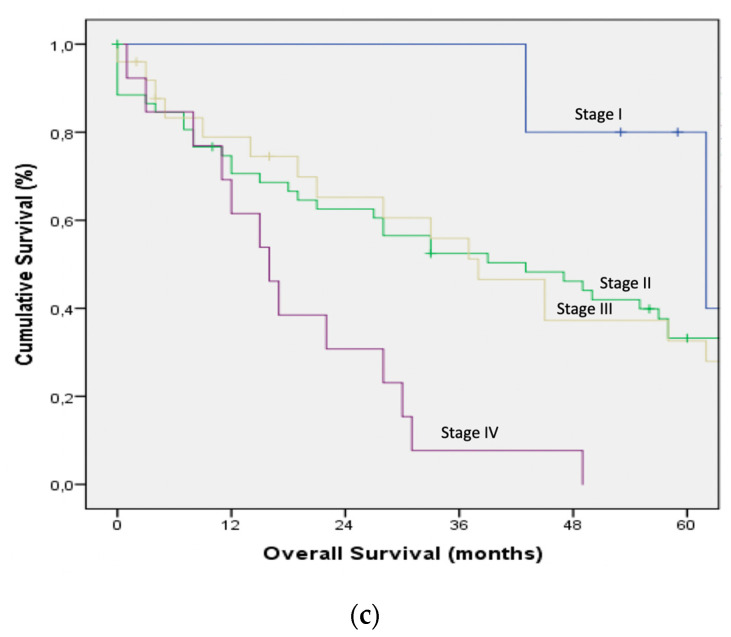
(**a**) Overall survival by the main stages according to the 6th edition of the American Joint Committee on Cancer staging system. (**b**): Overall survival by the main stages according to the 7th edition of the American Joint Committee on Cancer staging system. (**c**). Overall survival by the main stages according to the 8th edition of the American Joint Committee on Cancer staging system.

**Figure 2:**
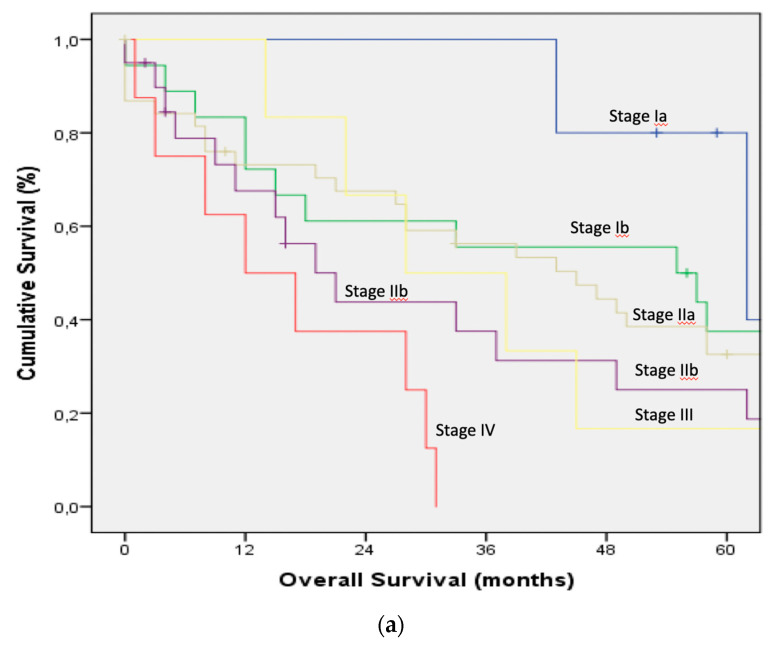

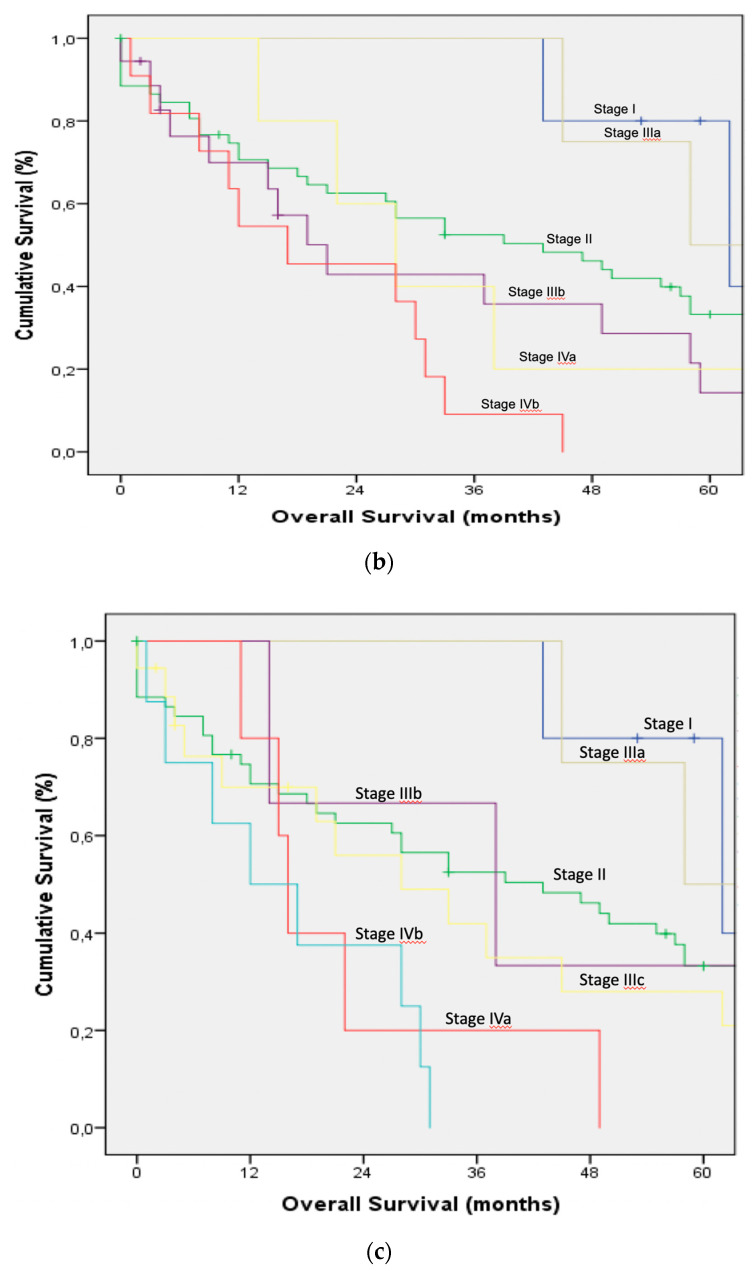
(**a**): Overall survival by the substages according to the 6th edition of the American Joint Committee on Cancer staging system. (**b**): Overall survival by the substages according to the 7th edition of the American Joint Committee on Cancer staging system. (**c**). Overall survival by the substages according to the 8th edition of the American Joint Committee on Cancer staging system.

Survival curves for the main and corresponding substages of the 6th, 7th, and 8th TNM editions were illustrated in Figure 1 and Figure 2.

According to the 6th edition AJCC TNM stage, five-year OS rates of 80% and 37.5% were observed in patients classified as stages IA and IB, respectively, whereas the survival of patients classified as stages IIA and IIB were similar, with five-year OS rates of 32.6% and 25%, respectively. Notably, patients in stage III had a five-year OS rate of 16.7%. None of the patients in stage IV survived five years after operation.

When patients were categorized by the 7th TNM edition, five-year OS rates were 80%, 33.2%, and 20.0% in stages I, II, and Iva, respectively. Notably, patients in stages IIIa and IIIb had five-year OS rates of 50% and 14.3%, respectively (Table 6). However, none of the patients categorized as stage IVb survived five years after operation.

Considering the 8th TNM edition of the AJCC staging, similar five-year OS rates were revealed for patients in stages I and II of 80% and 33.2%, respectively, compared to the survival rates for the 7th AJCC edition. Patients in stages IIIb and IIIc had comparable five-year OS rates of 33.3% and 28%, respectively, whereas the five-year OS rate of stage II was worse than stage IIIa (50%). In contrast, none of the patients in stage IV (a or b) were alive five years after liver resection.

After use of the 6th TNM edition, no increased risk of death was observed in patients classified in stages Ib, II (a and b), and III (all *p* > 0.05) compared with patients in stage Ia. By contrast, patients in stage IV had the greatest risk of death (stage IV vs. stage Ia, HR 8.41, 95% CI 1.74–40.57, *p* < 0.01).

Based on the AJCC 7th edition, patients defined as stages II, IIIa, IIIb, and IVa (all *p* > 0.05; Table 6) did not have higher risks of death compared with stage I patients. As expected, those in stage IVb had the greatest risk of death (stage IVb vs. stage I, HR 7.05, 95% CI 1.54–32.36, *p* = 0.01).

Compared with AJCC 8th edition stage I, patients in the 8th edition stages II, IIIa, IIIb, and IIIc (*p* > 0.05) revealed no increased risks of death. However, patients classified as stage IVa and IVb (stage IVa vs. stage I, HR 6.36, 95% CI 1.22–33.24, *p* = 0.028 and stage IVb vs. stage I, HR 8.66, 95% CI 1.79–41.83, *p* < 0.01) showed the greatest risks of death.

### 3.6. Prognostic Performance

The prognostic performance of the different staging systems was assessed by using ROC analysis and the Hoshmer-Lemeshow test (HLT) (Table 7).

In this context, similar results across the three analyzed AJCC staging systems could be observed. The 8th edition of the AJCC staging system showed greater discriminatory power with regards to the main tumor stages than the 7th edition (AUC: 8th edition vs. 7th edition: 0.66 vs. 0.61) but did not reach statistical significance.

Moreover, when expanding the 7th edition to include substages, its prognostic predictability slightly diminished (AUC: from 0.61 to 0.58), whereas with the expansion of the 8th edition with substages, the accuracy could be increased (AUC main stages vs. substages: 0.66 to 0.69) with a statistical trend (*p* = 0.09).

With regards to calibration of the evaluated staging systems, the HLT showed acceptable calibration and fitness of the analyzed 7th and 8th staging systems (Table 7).

## 4. Discussion

The management of PHC is challenging, and only selected patients are candidates for curative-intent surgery [30,31]. Therefore, accurate staging with a standardized staging system is essential not only for planning the frequency of post-operative surveillance, making therapeutic decisions, and/or selecting patients for adjuvant treatment but, also, for informing both patients and physicians about long-term clinical outcomes and for allowing for a standardized exchange of information about the extent of the disease [1,2]. While the optimal method for the risk stratification of patients with PHC remains unclear, the TNM system provided by the AJCC and the International Union Against Cancer (UICC) is the most commonly used method for stratifying outcomes among cancer patients [15,20,24,32].

In this context, the new 8th edition of the AJCC staging system has introduced four major changes (Table 1) and has especially included the recommended modifications by Ebata et al. [15], who underwent a survival analysis of 1399 patients undergoing liver resection for PHC at eight Japanese liver centers after modification of the T- and N- categories of the previously 7th TNM edition [15]. Based on these data, the authors could show that survival was slightly better for patients with advanced disease (T4 tumors, stage IVa) than for patients with regional lymph node-positive disease (stage IIIb), a finding that could be confirmed in our study. However, as already recognized, and as previous data have shown, marked differences exist in peri- and postoperative strategies and outcomes between Eastern and Western centers; therefore, external validation of the prognostic accuracy of staging systems in Western patients is essential [33,34].

A recently published study from two Western hepatobiliary centers evaluated the prognostic accuracy of the 8th TNM classification of the AJCC staging system in a cohort of 214 patients undergoing liver resection for PHC. In that study population, about 40% of the patients changed their stages from the 7th to the 8th AJCC edition. The authors determined that the new 8th TNM edition was only slightly better than the previous 7th edition [25].

Our analysis confirmed findings that could validate the new 8th TNM classification. For example, we observed a reclassification in a total of 25 patients (26.3%) when considering the substages (e.g., stage IIIa and IIIb) and in 23 patients (25%) when considering only the major stages (i.e., stage I, II, III, or IV). However, these modifications and consecutive reclassifications failed to provide any significant improvement in prognostic accuracy. Consequently, comparison of the prognostic accuracy of the 7th and 8th editions of the AJCC staging system using our results paralleled the findings of the study by Ruzzenente et al. [25]., wherein the 8th edition had only a slightly better discriminatory ability and power, with an AUC of 0.69 compared to 0.58 for the 7th edition. Nevertheless, the performance and ability to stratify patient prognosis of the newly released 8th edition was still unsatisfactory (AUC < 0.7).

The extent of intrahepatic biliary invasion is a critical factor that determines surgical indications and the type of hepatectomy that should be performed in patients with PHC. To ensure a curative liver resection (R0 margins) in patients with Bismuth type II and III PHC, the resection should include a hemihepatectomy combined with en-bloc resection of the first segment (caudate lobe), as well as an extrahepatic bile duct resection to ensure negative resection margins [25,29]. However, in patients with Bismuth type IV disease (characterized by having bilateral involvement of the second-order intrahepatic bile ducts), the standard operative approach remains unclear, and many centers still consider Bismuth type IV tumors unresectable [2,35,36,37,38]. Conversely, as surgical techniques and perioperative management have improved, some surgeons have suggested that a type IV tumor does not always imply unresectability [6,15,35,39]. In this context, these surgeons have proposed a more aggressive operative approach for patients with a Bismuth type IV PHC, including an extended hepatectomy combined with caudate lobectomy, extended lymph node dissection, and vascular resection with portal vein and/or hepatic artery reconstruction, to achieve negative resection margins [3,8,15,35,40,41]. The results of previous studies indicate that this aggressive strategy could further improve long-term outcomes and prolong patient survival; therefore, this procedure could be established as a state-of-the-art operation for patients with locally advanced PHC and, especially, for Bismuth-Corlette types III-IV hilar cholangiocarcinomas [3,34,41,42].

The Japanese group associated with Ebata et al. [15] was the first to report that patients with pN0M0 disease who underwent an R0 liver resection showed no significant differences in survival when compared to patients with Bismuth type I/II, III, and IV tumors, as the five-year OS rates were 63.1%, 65.6%, and 59.2%, respectively (*p* > 0.05). Similar findings were obtained in a recently published study by Ruzzenente et al. [25], as well as in the present study, as the five-year survival rate was 50.1% in our 30 patients with pN0M0 who underwent R0 resection for a Bismuth Type IV PHC. Although these patients represent a highly selected subset of patients undergoing aggressive surgery, these findings nevertheless suggest that an aggressive operative approach with or without vascular resection could offer a curative option even for patients with Bismuth type IV PHC [15,25,36]. Furthermore, the T staging should separate the longitudinal extension of the disease from its vascular invasion.

Collectively, these data suggest that, while PHC generally has a poor prognosis, large differences in survival can be observed among patients based on a subset of clinical and pathological factors. In our cohort of patients, several factors, including gender, age, surgical radicality, the extent of liver resection (± caudate lobe resection), tumor differentiation, and distant metastases (e.g., lymph node status), were independent prognostic factors for long-term survival, in agreement with previous observations [12,42,43].

In this context, the prognostic impact of the LN status is considered as one of the most important factors for long-term survival following liver resection for PHC [5,18,44,45,46]. A certain benchmark number of retrieved lymph nodes is necessary to secure a representative and adequate staging. However, the extent of the lymphadenectomy during the resection of PHC, such as the minimum number of LNs to be retrieved, remains under controversial debate, and marked differences in the operative approach between Western and Eastern centers have been reported [44,45,47,48]. Recent SEER register analyses showed that the retrieved LN counts are independent prognostic survival factors for node-negative and node-positive PHC patients [49,50]. Therefore, an insufficient LN count may result in understaging of the extent of disease, and a subsequent poor recovery of LNs may increase the rate of incorrectly classified N0 patients and overestimate the survival expectancy.

The required extent of lymphadenectomy for patients with PHC remains controversial, especially with respect to the minimum number of LNs that should be examined. The latest TNM classification recommended a LN count greater than or equal to 15 LNs as a requirement for adequate LN staging. Since then, a wave of criticisms emerged among experts, and some retrospective and prospective studies examined several different LN staging systems in an attempt to obtain a better prediction of the survival of patients with gastrointestinal tumors [44,45,46,47,48,51,52]. The authors of a previously published systemic review of 20 studies suggested that a LN count greater than or equal to seven is adequate for prognostic staging, while a LN count greater than or equal to 15 does not improve detection of patients with positive LNs [44]. By contrast, a recently published international, multi-center study that included 437 patients with PHC who underwent liver resection reported a required median number of retrieved LNs of three (IQR one–seven), and the incidence of LN metastasis was 36%, similar to other published values from Western centers [47]. In another recently published study by Ruzzenente et al., the median number of harvested lymph nodes was five (IQR 3-10), with an incidence of lymph node metastases in 45% of the patients. However, the number of harvested LNs did not have an impact on patient prognosis in uni- or multivariate analyses [25].

We found similar results in our study, where extensive lymphadenectomy was performed in almost all patients (*n* = 91 patients, 96%) undergoing a curative-intent hepatectomy. In up to 48% of patients, more than five LNs were retrieved. The number of LNs harvested did not have an impact on patient survival, but the multivariate survival analysis confirmed that the number of MLNs was strongly associated with prognosis (1–3 MLN vs. 0 MLN, HR = 1.7, 95% CI: 1.1–2.8; *p* = 0.04 and > 3 MLN vs. 0 MLN, HR = 3.2, 95% CI: 1.2–8.3; *p* = 0.01). Notably, no patients with more than three metastatic lymph nodes (N2, according to the AJCC 8th edition N staging) survived five years after operation, compared with a five-year OS of 24.1% for patients with one–three metastatic lymph nodes (N1, according to the 8th edition; data not shown).

### Limitations of the Study

The results of the current study should be interpreted while considering several limitations. One limitation is that, although our study is surely one of the larger Western series of patients with resected PHC to be reported in the literature, our sample size is nevertheless still insufficient, especially with a small number of patients in stage I (7th and 8th editions; *n* = 5 patients) and stage IV (7th edition: *n* = 16 patients and 8th edition: *n* = 13 patients), to allow the drawing of definitive conclusions and derive statistical power about small modifications in this staging system. The retrospective nature of our study and the long study time (over 17 years) presents a risk of selection and confounding biases regarding the diagnosis and treatment options of the patients. Therefore, larger cohorts of patients are needed to test the TNM classification, although this is challenging, given the rare incidence of PHC.

A second limitation is our inclusion of only patients with resectable disease who underwent a liver resection. The TNM classification offers a precise tumor-staging algorithm aside from the treatment set and is widely used in cancer staging. However, only surgery and pathological examinations are able to provide an accurate confirmation of the TNM stages. The advantage of our study is that pathological confirmation of the TNM stage was available for all patients. The disadvantage is that the observed results cannot be extrapolated to patients who did not undergo resection because of extrahepatic metastasis or locally advanced tumor disease. However, we included only resected patients, because the assessment of both the T stage and N stage is inaccurate without the microscopic evaluation of a resected specimen. In this context, the accuracy of the estimation of the nodal status based on an evaluation of the size criteria on cross-sectional imaging is very limited. For example, Ruys et al. [53] showed sensitivity and specificity estimates of 61% and 88% for lymph node involvement based on the CT staging of patients with PHC. Furthermore, only 37% of the lymph nodes >30mm in diameter were confirmed as positive after analyzing slides of 147 patients undergoing liver resection for PHC. These findings indicate a very low diagnostic accuracy of cross-sectional imaging for the staging of perihilar cholangiocarcinoma and, especially, for nodal involvement [53].

A third limitation is that our study included patients undergoing nonradical (R1) and suboptimal tumor resections (distal bile duct resections and no caudate lobe resections) and/or patients with involvement of the main portal vein or common hepatic artery. The improvements in the standard operative and perioperative approaches, in combination with new neoadjuvant or adjuvant chemotherapeutic agents, necessitates the inclusion of sensitivity analyses of those patients who underwent R0-extended hepatectomy with caudate lobe resections to compare them with the whole cohort of patients.

Lastly, the number of harvested and examined lymph nodes in the final pathology varied from 1 to 20 (median four with IQR 3-8; mean number 6.1 ± 4.3). There were 18 patients (19%) with less than three LNs examined; the possibility of understaging cannot be ruled out, and further analyses would be needed.

## 5. Conclusions

An optimal staging system should provide information about the prognosis, guide the therapy, and allow comparisons with different staging systems. However, none of the currently existing PHC staging systems fulfills these criteria. Historically, the AJCC staging is based mainly on the anatomic extent of the tumor. Although nonanatomic factors have been introduced into the staging of some cancers (e.g., mitotic rate in melanoma), with reference to PHC, the 6th, 7th, and 8th editions of the AJCC staging system have all adhered to the anatomic extent of the tumor, as in all other hepatobiliary and pancreatic cancers [11,23]. Despite recently encouraging results reported by Ebata et al. at a large Eastern hepatobiliary center, who attempted to improve the staging and prognosis of PHC patients, the results of our retrospective study of 95 resected PHC patients indicate that the newly released 8th edition of the AJCC staging system does not provide a better ability to stratify the prognosis and predict the clinical outcomes of patients with PHC when compared with the previous 6th and 7th editions.

New advances in genomic and transcriptomic profiling have contributed to a better understanding of the genetic landscape of molecular alterations in PHC and offer hope for the development of novel targeted therapies [54,55,56]. Future randomized clinical controlled trials are needed to focus on the targeting of deregulated signaling pathways, with the goal of personalizing treatments for patients with PHC and for the prospective assignment of patients based on their transcriptomic and genetic profiles. In this context, this new information, in combination with other clinicopathologic features (i.e., extended negative margins, lymph node status, and the use of neoadjuvant and adjuvant chemotherapies), might improve our ability to predict the prognosis of patients with PHC. Nevertheless, for outcome comparisons, future refinements are needed to improve the staging of patients with PHC. Future research should therefore investigate whether a combination of AJCC staging and nonanatomic independent prognostic factors can further improve individual patient prognosis.

## Figures and Tables

**Table 1:** American Joint Committee on Cancer (AJCC) staging system by tumor-node-metastasis (TNM) stage of the 6th, 7th, and 8th editions.

	6th Edition		7th Edition		8th Edition
**T0**	No evidence of primary tumor		No evidence of primary tumor		No evidence of primary tumor
**Tis**	Carcinoma in situ		Carcinoma in situ		Carcinoma in situ
**T1**	Tumor confined to the bile duct		Tumor confined to the bile duct, with extension up to the muscle layer or fibrous tissue		Tumor confined to the bile duct, with extension up to the muscle layer or fibrous tissue
**T2**	Tumor invades beyond the wall of the bile duct				
**T2a**			Tumor invades beyond the wall of the bile duct to surrounding adipose tissue		Tumor invades beyond the wall of the bile duct to surrounding adipose tissue
**T2b**			Tumor invades adjacent hepatic parenchyma		Tumor invades adjacent hepatic parenchyma
**T3**	Tumor invades the liver, gall bladder, pancreas, and or unilateral branches of the portal vein (right or left) or hepatic artery (right or left)		Tumor invades unilateral branches of the portal vein or hepatic artery		Tumor invades unilateral branches of the portal vein or hepatic artery
**T4**	Tumor invades any of the following: main portal vein or its branches bilaterally, common hepatic artery, or other adjacent structures, e.g., colon, stomach, duodenum, and abdominal wall		Tumor invades the main portal vein or its branches bilaterally, or the common hepatic artery, or the second-order biliary radicals bilaterally, or unilateral second-order biliary radicals with contralateral portal vein or hepatic artery involvement		Tumor invades the main portal vein or its branches bilaterally, or the common hepatic artery, or unilateral second order biliary radicals with contralateral portal vein or hepatic artery involvement
**NX**	Regional lymph nodes cannot be assessed		Regional lymph nodes cannot be assessed		Regional lymph nodes cannot be assessed
**N0**	No regional lymph node metastasis		No regional lymph node metastasis		No regional lymph node metastasis
**N1**	Regional lymph node metastases are the cystic duct, pericholedochal, hilar, peripancreatic (head only), periduodenal, periportal, celiac, and superior mesenteric nodes		Regional lymph node metastasis including nodes along the cystic duct, common bile duct, common hepatic artery, and portal vein		Metastasis to 1–3 regional lymph nodes
**N2**					Metastasis to 4 or more regional nodes
**MX**	Distant metastasis cannot be assessed		Distant metastasis cannot be assessed		
**M0**	No distant metastasis		No distant metastasis		No distant metastasis
**M1**	Distant metastasis		Distant metastasis		Distant metastasis
**American Joint Committee on Cancer (AJCC) staging system.**					
	**AJCC, 6th Edition**		**AJCC, 7th Edition**		**AJCC, 8th Edition**
**Stage 0**	Tis→N0→M0	Stage 0	Tis→N0→M0	Stage 0	Tis→N0→M0
		Stage I	T1→N0→M0	Stage I	T1→N0→M0
**Stage IA**	T1→N0→M0				
**Stage IB**	T2→N0→N0				
		Stage II	T2a,b→N0→M0	Stage II	T2a,b→N0→M0
**Stage IIA**	T3→N0→M0				
**Stage IIB**	T1–3→N1→M0				
**Stage III**	T4→AnyN→M0				
		Stage IIIA	T3→N0→M0	Stage IIIA	T3→N0→M0
		Stage IIIB	T1–3→N1→M0	Stage IIIB	T4→N0→M0
				Stage IIIC	T1-4→N1→M0
**Stage IV**	T1–4→AnyN→M1				
		Stage IVA	T4→AnyN→M0	Stage IVA	T1-4→N2→M0
		Stage IVB	T1–4→AnyN→M1	Stage IVB	T1-4→AnyN→M1

**Table 2:** Demographic and clinicopathologic characteristics of 95 patients with resected perihilar cholangiocarcinoma. IQR: interquartile range.

Variables	Patients (%)
**Age** (years), median (SD)	64.9 ± 10.2
**Bismuth classification**	
I	6 (6.3)
II	8 (8.4)
IIIa	11 (11.6)
IIIb	12 (12.6)
IV	49 (51.6)
N/A	9 (9.5)
**Gender**	
Male	52 (54.7)
Female	43 (45.3)
**Type of hepatectomy**	
Right hepatectomy	10 (10.5)
Left Hepatectomy	11 (11.6)
Extended right hepatectomy	54 (56.8)
Extended left hepatectomy	16 (16.8)
Other	4 (4.4)
**Margin status**	
R0	68 (71.6)
R1	27 (28.4)
**Tumor differentiation**	
Well (G1)	5 (5.3)
Moderately (G2)	42 (44.2)
Poor (G3)	48 (50.5)
**Lymph node status**	
Negative	60 (63.2)
Metastatic	27 (28.4)
N/A	8 (8.4)
**Distant metastasis**	
No	86 (90.5)
Yes	9 (9.5)
**Vascular invasion**	
No	63 (66.3)
Yes	24 (25.3)
N/A	8 (8.4)
**Perineural invasion**	
No	17 (17.9)
Yes	59 (62.1)
N/A	19 (20.0)
**Invasion of small lymphatic vessels**	
No	20 (21.0)
Yes	68 (71.6)
N/A	7 (7.4)
**Adjuvant chemotherapy**	
No	69 (72.7)
Yes	26 (27.3)
**Portal vein invasion**	
No	87 (91.6)
Yes	8 (8.4)
**Portal vein embolization**	
No	74 (77.9)
Yes	21 (22.1)
**Preoperative bilirubin (µmmol/l), mean (SD)**	44.2 (11.1)
**Preoperative drainage**	
No	17 (17.9)
PTCD	9 (9.5)
ERCP	61 (64.2)
both	3 (3.2)
N/A	5 (5.3)
**Harvested lymph node, median (IQR)**	4 (3–8)
**Metastatic lymph node, median (IQR)**	1 (0–2)
**Portal vein resection**	
Yes	64 (67.3)
No	21 (32.7)
**Caudate resection**	
Yes	67 (70.5)
No	28 (29.5)

**Table 3:** Cross-tabulation of the 6th, 7th, and 8th editions of the American Joint Committee on Cancer (AJCC) staging system.

		**Stage 7**							
**Stage 6**		**I**	**II**	**IIIa**	**IIIb**	**IVa**	**IVb**	**Total**	
	Ia	5						**5**	
	Ib		18					**18**	
	IIa		34	4				**38**	
	IIb				18		2	**20**	
	III					5	1	**6**	
	IV						8	**8**	
		**5**	**52**	**4**	**18**	**5**	**11**	**95**	
		**Stage 8**							
**Stage 6**		I	II	IIIa	IIIb	IIIc	IVa	IVb	**Total**
	Ia	5							**5**
	Ib		18						**18**
	IIa		34	4					**38**
	IIb					16	4		**20**
	III				3	2	1		**6**
	IV							8	**8**
		**5**	**52**	**4**	**3**	**18**	**5**	**8**	**95**
		**Stage 8**							
**Stage 7**		I	II	Ia	IIIb	IIIc	IVa	IVb	**Total**
	I	5							**5**
	II		52						**52**
	IIIa			4					**4**
	IIIb					15	3		**18**
	IVa				3	1	1		**5**
	IVb					2	1	8	**11**
		**5**	**52**	**4**	**3**	**18**	**5**	**8**	**95**

**Table 4:** Univariate Kaplan-Meier survival analysis of the study population (*n* = 95 patients).

Variables	Patients (%)	Median Survival (Months, 95%CI)	Log-Rank Test (*p*-Value)
**Age**			
≤65	47 (49.5%)	43 (1.9–40.1)	0.03
>65	48 (50.5%)	21 (17.4–68.5)	
**Bismuth-Classification**			
I/II	14 (14.7)	56 (20.9–91.1)	0.772
III/IV	72 (75.8)	33 (15.2–50.7)	
N/A	9 (9.5)		
**Gender**			
Male	52 (54.7%)	19 (11.3–26.8)	0.02
Female	43 (45.3%)	56 (37.1–74.9)	
**Residual tumor**			
R0	68 (71.6)	45 (24.7–65.2)	0.003
R1	27 (28.4)	13 (5.1–23.1)	
**Tumor differentiation**			
Well/moderately (G1/G2)	47 (49.5)	47 (27.3–66.8)	0.01
Poor (G3)	48 (50.5)	15 (3.8–26.1)	
**Lymph node status**			
Negative	60 (63.2)	50 (28.5–71.5)	0.004
Metastatic	27 (28.4)	11 (4.9–17.1)	
N/A	8 (8.4)		
**Harvested Lymph node**			
<5	50 (51.6)	43 (25.7–60.6)	0.656
>5	45 (48.4)	29 (8.4–41.3)	
**Distant metastasis**			
No	86 (90.5)	41 (26.1–59.8)	<0.001
Yes	9 (9.5)	8 (7.1–8.9)	
**Vascular invasion**			
No	63 (66.3)	37 (21.5–52.5)	0.006
Yes	24 (25.3)	12 (5.3–18.7)	
N/A	8 (8.4)		
**Perineural invasion**			
No	17 (17.9)	57 (8.3–105.6)	0.678
Yes	59 (62.1)	21 (7.4–34.5)	
N/A	19 (20.0)		
**Invasion of small lymphatic vessels**			
No	20 (21.1)	56 (8.7–103.3)	0.139
Yes	68 (71.6)	20 (6.6–33.3)	
N/A	7 (7.4)		
**Adjuvant chemotherapy**			
No	77 (81.1)	27 (1.4–52.8)	0.780
Yes	18 (18.9)	38 (28.4–47.6)	
**Portal vein invasion**			
No	87 (91.6)	27 (21.9–52.1)	0.899
Yes	8 (8.4)	20 (0.1–44.9)	
**Portal vein embolization**			
No	74 (77.9)	38 (22.1–53.9)	0.849
Yes	21 (22.1)	21 (1.4–43.9)	
**Preoperative drainage**			
No	17 (17.9)	19 (0.1–46.7)	0.194
Yes	73 (76.8)	37 (16.8–57.2)	
N/A	5 (5.3)		
**Caudate resection**	67 (70.5%)		0.004
Yes	28 (29.5%)	47 (26.8–67.2)	
No		11 (4.5–17.5)	
**Portal Vein Resection**	64 (67.3%)		0.069
Yes	21 (32.7%)	45 (6.5–23.4)	
No		15 (18.9–71.1)	

**Table 5:** Multivariate survival analysis—Cox model—overall survival (*n* = 95 patients).

Variables	Hazard Ratio (95% CI)	*p*-Value
**Age** (years)		
≤65		
>65	1.9 (1.2–3.2)	0.040
**Residual tumor**		
R0		
R1	2.1 (1.2–3.5)	0.01
**Tumor differentiation**		
G1		-
G2	5.7 (0.8–42.3)	0.08
G3	7.9 (1.1–58.1)	0.04
**Metastatic Lymph Node**		
0		-
1–3	1.1 (0.5–2.1)	0.04
>3	3.5 (1.4–9.1)	0.009
**Distant metastasis**		
Yes	4.2 (1.7–10.8)	0.002
No		
**Vascular invasion**		
Yes	1.68 (0.9–3.1)	0.10
No		
**Caudate Resection**		
Yes	2.2 (1.2–4.1)	0.007
No		
**Gender**		
Male	2.63 (1.4–4.9)	0.003
Female		

**Table 6:** Stage-specific survival rates due to the different TNM editions of the AJCC staging system.

**Subgroup stage-specific patient survival according to the 6th, 7th, and 8th editions of the American Joint Committee on Cancer (AJCC) staging system of our study population (*n* = 95 patients).**								
	**Number Patients,** **% (*n*)**	**1-Year-Survival, %**	**3-Year-Survival, %**	**5-Year-Survival,** **%**	**Median Survival,** **Months (95% CI)**	**Hazard Ratio** **(95% CI)**	**Cox Regression** **(*p*-Value)**	**Log-Rank** **(*p*-Value)**
**Overall**	100 (95)							
**6th Edition**								0.035
I	24.2 (23)							
a	5.3 (5)	100.0	100.0	80.0	62 (34.4–89.6)		0.040	
b	18.9 (18)	72.2	55.6	37.5	55 (8.0–102.0)	2.66 (0.60–11.72)	0.196	
II	61.1 (58)							
a	40.0 (38)	73.2	56.3	32.6	45 (25.6–64.4)	2.42 (0.57–10.26)	0.229	
b	21.2 (20)	67.6	37.5	25	21 (11.6–30.4)	3.67 (0.83–16.20)	0.086	
III	4.3 (6)	100.0	50.0	16.7	28 (8.8–47.2)	3.30 (0.64–17.08)	0.155	
IV	8.4 (8)	50.0	-	-	12 (0.0–24.5)	8.41 (1.74–40.57)	0.008	
**7th Edition**								0.012
I	5.3 (5)	100.0	100.0	80.0	62 (34.4–89.6)		0.037	
II	54.7 (52)	70.6	52.5	33.2	43 (19.5–66.5)	2.67 (0.64–11.09)	0.177	
III	23.2 (22)							
a	4.2 (4)	100.0	100.0	50.0	58 (51.8–87.5)	1.19 (0.17–8.47)	0.860	
b	18.9 (18)	69.9	42.9	14.3	21 (12.2–29.7)	3.42 (0.76–15.34)	0.108	
IV	16.8 (16)							
a	5.3 (5)	100.0	40.0	20.0	28 (15.1–40.9)	3.29 (0.60–18.07)	0.170	
b	11.6 (11)	54.5	9.1	-	17 (0.0–35.3)	7.05 (1.54–32.36)	0.012	
**8th Edition**								0.009
I	5.3 (5)	100.0	100.0	80.0	62 (34.4–89.6)		0.023	
II	54.7 (52)	70.6	52.5	33.2	43 (19.5–66.5)	2.68 (0.64–11.14)	0.175	
III	26.3 (25)							
a	4.2 (4)	100.0	100.0	50.0	58 (51.8–87.5)	1.19 (0.17–8.46)	0.861	
b	3.2 (3)	100	66.7	33.3	38 (00.0–76.4)	2.32 (0.33–16.52)	0.400	
c	18.9 (18)	69.9	42.2	28.0	28 (6.4–49.6)	3.30 (0.74–14.80)	0.119	
IV	13.7 (13)							
a	5.3 (5)	80.0	20.0	-	16 (13.9–18.1)	6.36 (1.22–33.24)	0.028	
b	8.4 (8)	50.0	-	-	12 (00.0–24.5)	8.66 (1.79–41.83)	0.007	
**Main-group stage-specific patient survival according to the 6th, 7th, and 8th editions of the American Joint Committee on Cancer (AJCC) staging system of our study population (*n* = 95 patients).**								
	**Number Patient,** **% (*n*)**	**1-Year-Survival, % (*n*)**	**3-Year-Survival, % (*n*)**	**5-Year-Survival,** **% (*n*)**	**Median Survival,** **Months (95% CI)**	**Hazard-Ratio** **(95% CI)**	**Cox Regression** **(*p*-Value)**	**Log-Rank** **(*p*-Value)**
**Overall**	95	68.4	46.1	36.2	21 (8.1–33.9)			
**6th Edition**								0.039
I	24.2 (23)	78.3	65.2	46.0	58 (48.8–67.2)		0.027	
II	61.1 (58)	71.4	50.2	30.1	37 (21.4–52.6)	1.25 (0.70–2.25)	0.450	
III	4.3 (6)	100.0	50.	16.7	28 (8.8–47.2)	1.49 (0.54–4.09)	0.438	
IV	8.4 (8)	50.0	-	-	12 (0.0–24.5)	3.76 (1.55–9.13)	0.003	
**7th Edition**								0.01
I	5.3 (5)	100.0	100.0	80.0	62 (34.4–89.6)		0.016	
II	54.7 (52)	70.6	52.5	33.2	43 (19.5–66.5)	2.65 (0.64–11.03)	0.180	
III	23.2 (22)	75.8	54.7	32.8	37 (1.6–72.4)	2.68 (0.61–11.83)	0.192	
IV	16.8 (16)	68.8	18.8	6.3	22 (7.6–36.4)	5.27 (1.20–23.36)	0.018	
**8th Edition**								0.002
I	5.3 (5)	100.0	100.0	80.0	62 (34.4–89.6)		0.005	
II	54.7 (52)	70.6	52.5	33.2	43 (19.5–66.5)	2.67 (0.64–11.09)	0.177	
III	26.3 (25)	78.9	55.9	32.6	38 (24.6–51.4)	2.58 (0.59–11.23)	0.207	
IV	13.7 (13)	61.5	7.7	-	16 (10.1–21.9)	7.47 (1.65–33.74)	0.009	

**Table 7:** Discrimination power and calibration of the 6th, 7th, and 8th editions of the American Joint Committee on Cancer (AJCC) staging system. AUC: area under the curve.

Edition	AUC (95% CI)	*p*-Value	Calibration *χ*^2^	*p*-Value
AJCC 6th edition—main stages	0.54 (0.43–0.67)	0.42	5.1	0.05
AJCC 6th edition—substages	0.56 (0.42–0.69)	0.32	9.2	0.11
AJCC 7th edition—main stages	0.61 (0.51–0.73)	0.17	1.1	0.27
AJCC 7th edition—substages	0.58 (0.46–0.71)	0.19	2.8	0.21
AJCC 8th edition—main stages	0.66 (0.45–0.79)	0.14	2.1	0.31
AJCC 8th edition—substages	0.69 (0.52–0.84)	0.09	1.6	0.39

CI, confidence interval.

## Data Availability

Our database contains highly sensible data that may provide insight into clinical and personnel information about our patients and lead to identification of these patients. Therefore, according to organizational restrictions and regulations, these data cannot be made publicly available. However, the datasets used and/or analyzed during the current study are available from the corresponding author on reasonable request.

## Abbreviations

PHC	Perihilar cholangiocarcinoma
AJCC	American Joint Committee on Cancer
TNM	tumor node metastasis
HPB	hepato-pancreato-biliary
CT	computed tomography
MRI	magnetic resonance imaging
MRCP	magnetic resonance cholangiopancreatography
EBD	Endoscopic biliary drainage
PTBD	Percutaneous transhepatic biliary drainage
PVE	portal vein embolization
CLR	caudate lobe resection
ASA	American Society of Anesthesiologists
CEA	carcinoembryonic antigen
CA	Cancer Antigen
IQR	interquartile range
ANOVA	Analysis of variance
OS	overall survival
HR	hazard ratio
CI	confidence interval
AUC	Area under the curve
ROC	Receiver operating characteristic curve
PVR	Portal vein resection
LN	Lymph node
MLN	Metastasic lymph node
HLT	Hoshmer-Lemeshow test
UICC	International Union Against Cancer
